# New wireless architectures based on information metasurfaces

**DOI:** 10.1093/nsr/nwad013

**Published:** 2023-01-11

**Authors:** Vincenzo Galdi

**Affiliations:** Fields & Waves Lab, Department of Engineering, University of Sannio, Italy

Mobile communications have come a long way since the early days of cellular networks. Today, we are on the cusp of a new generation of wireless technology, known as sixth-generation (6G), which is expected to significantly improve upon the performance, efficiency and functionality of its predecessor, 5G. While 5G has already begun to transform the way we connect and communicate, 6G is poised to take these capabilities to the next level, with significant impact on industry and society [1].

Specifically, key figures of merit that are targeted for 6G include data rates of up to 1 Tbps, latencies of <1 ms and energy efficiencies up to 100% higher than 5G. These improvements will enable a wide range of new applications and use cases that require enhanced communication capabilities, including those involving the ‘internet of everything’, intelligent transportation systems, truly immersive extended reality, high-fidelity mobile holograms and digital replicas, among others.

Significant advancements like those anticipated with 6G require a fundamental shift in approach. Smart radio environments [2], which enable adaptable communication systems that can adjust to changing conditions, and emerging technologies like metasurfaces, specifically ‘reconfigurable intelligent surfaces’ [3], are expected to be crucial in this respect. These technologies can overcome challenging propagation environments and create highly directional, efficient and environmentally responsive communication links. Moreover, they can enable simultaneous wireless information and power transmission [4], thereby greatly enhancing the energy sustainability.

**Figure 1.**
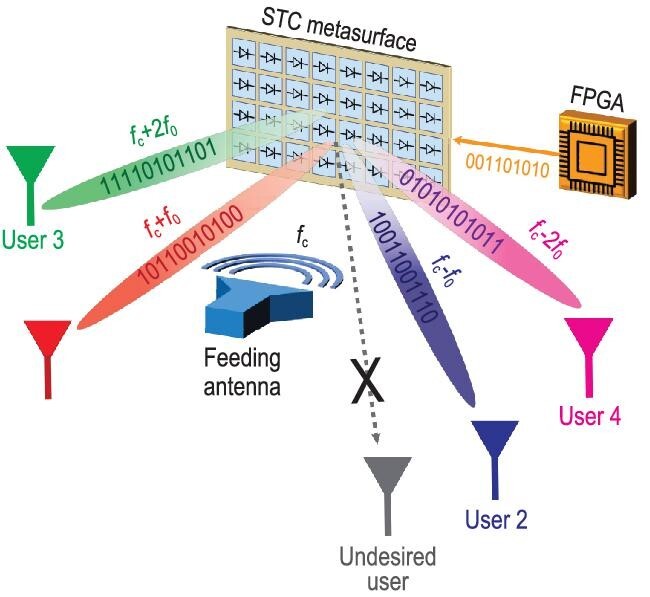
Conceptual illustration of space- and frequency-division multiplexing enabled by STC metasurfaces.

Recent research led by Tie Jun Cui, Qiang Cheng and Shi Jin at Southeast University [5] has demonstrated the possibility to enable space- and frequency-division multiplexing capabilities in these platforms, without the need for complex and bulky radio-frequency components such as digital-to-analog converters and mixers. As conceptually illustrated in Fig. 1, the proposed approach relies on a digital, programmable platform [6], composed of a 2D array of meta-atoms whose reflection responses can be independently switched between a series of discrete states (e.g. quantized reflection phases) by means of diodes controlled via a field-programmable gate array. This platform, which belongs to the general class of ‘information meta-structures’ [7], enables the realization of a large number of distinct functionalities and their switching in real time. Crucial to the proposed multiplexing concept is the introduction of *dynamic* modulation, with the switching controlled both spatially and temporally according to a suitably designed space-time coding (STC) [8]. This enables joint spatial-spectral field manipulations, whereby it is possible to generate multiple harmonic frequencies ${f}_c \pm m{f}_0$ (with ${f}_c$ and ${f}_0$ denoting the carrier and modulation frequencies, respectively, and *m* being an integer) exhibiting different, controllable scattering patterns. In the presence of multiple users located at different directions (see Fig. 1), by representing with the digits ‘1’ and ‘0’ a strong or weak signal (i.e. main or minimum scattering lobe, respectively) at a given harmonic frequency and direction, it becomes therefore possible to directly encode some digital information in a number of independent communication channels and exclude undesired users located at other directions, thereby realizing space- and frequency-division multiplexing in a rather simple, efficient and secure fashion. As a proof of concept, a dual-channel wireless communication system operating at X-band was fabricated and tested, demonstrating a transmission rate of 2.5 Mbps and low interference between different user channels [5].

The above results suggest that space-time-coding information metasurfaces are very promising candidates for use in smart radio environments for 6G communication systems. Future developments in this field may include the use of artificial intelligence for enhanced environmental responsiveness, the incorporation of nonreciprocal effects for full-duplex communication and faster modulation schemes for mm-wave and THz frequencies of potential interest for 6G technologies.

***Conflict of interest statement*.** None declared.
